# Amber Bases for Artificial Teeth

**Published:** 1859-12

**Authors:** George Dieffenbach

**Affiliations:** Surgeon Dentist, 389 Canal Street, New York


To the Editors of the New York Dental Journal:

Sirs: I beg to call your attention to the Amber Bases for Artificial Teeth, which are secured to me by three distinct Letters Patent, that is to say, by Letters Patent No. 19,916, dated April 13, 1858, by Letters Patent No. 24,544, dated June 28, 1859, and by Letters Patent No. 24,545, dated June 28, 1859.

The great usefulness of this invention for dental purposes, and its superiority over the existing methods of forming a base for artificial teeth, consist in the following points:—

1st. The Amber composition is tasteless; it causes no excitement of the glands; it creates an agreeable feeling in the mouth, and emits no odor.

2d. The contact between the Amber Composition and the mouth is not injurious to the health, and acts as a preventive against some diseases, such as inflammation of the throat, etc.

3d. Being a bad conductor of heat, it does not cause that certain feeling of chill in the mouth, which metal bases create.

4th. It is not liable to oxydization, corrosion or solution by the action of stomach acid or of saliva.

5th. It is capable of bearing an extreme degree of heat, without suffering any injury therefrom.

6th. It can be easily cast into any shape suitable to the mouth; it is not liable to bend; it always retains its form; and for these reasons it affords greater security for its retention in the mouth, and increased facilities in chewing.

7th. Being enabled to firmly unite the artificial teeth with this composition without the use of any other cement or solder, by uniting them before the composition becomes hardened, I obtain a compact body, and avoid porous between the teeth and their base.

8th. Being enabled to represent an exact imitation of flesh color in my amber compositions, I can produce compact, continuous, and homogeneous gums *without the aid of any gum teeth whatever.*

9th. There being no sulphur used in my composition, it is superior to vulcanized Rubber or Gutta Percha, as it emits no disagreeable odor.

10th. The profession is well aware of the fact, that vulcanized Rubber or gutta parcha bases represent so bad an imitation of flesh color that it is always necessary to represent the gum by a coating or lining of porcelain; and in this particular also my Amber Bases are superior.

These and other advantages will secure to this Amber Composition a preference over all other metallic or non-metallic bases, and I am confident that all professional dentists will find it advantageous for themselves and their Patients to employ amber, instead of metals, India Rubber or Gutta Percha.

To give a correct idea of the process and of the composition of matter constituting the new invention, I refer to the specifications annexed to the three patents above mentioned, and I will confine myself here to a statement of some of the principal points of the same.

The first Patent, No. 19,916, refers to the manner of compounding and shaping the amber composition, and a resume of the invention is thus expressed: "What I claim as new and desire to secure by Letters Patent is, making the base for artificial teeth of a composition of matter in which AMBER forms the principal ingredient, in the manner substantially as described."

The second Patent, No. 24,544, says: I add to one pound of amber, treated as described in my former Patent, about one-half pound of pulverized *Sulphate of Alumina*, about one-half pound of *purified Linseed oil*, about three-fourths of a pound of Gutta Percha or Caoutchouc, and about one-fourth of a pound of a mixture of metallic colors most nearly resembling the color of the natural healthy gum."

"The compound thus formed may consist of the following combinations: Sulphate of Alumina in combination with Amber, Caoutchouc or Gutta Percha, and linseed oil, with or without coloring matter; or Sulphate of Alumina in combination with amber and linseed oil, with or without coloring matter; or Sulphate of Alumina in combination with Caoutchouc or Gutta Percha, with or without coloring matter."

"What I claim herein as my invention and desire to secure by Letters Patent is, the composition of matter consisting of

Sulphate of Alumina and other ingredients, substantially as described for the purpose set forth."

My third Patent, No. 24,545, for an improvement in the coloring process, contains the following passages:

"My attention was first drawn to the necessity of providing some means for giving color to compounds intended to represent artificial gums by the bad representation of flesh color in the existing artificial gums. The compound termed "Coralite" consists of Gutta Percha, Sulphur, and some other coloring matter; another compound, termed "Vulcanite," consits of India Rubber, Sulphur, and coloring matter, but the dental plates thereby produced are so imperfect in regard to their color that it is generally necessary to coat them with porcelain in the same manner as metallic gums are coated, by which their weight is greatly increased and their shape impaired."

"What I claim as my invention and desire to secure by Letters Patent is, developing the color of a cured or hardened composition by the agency of solar light when the coloring matter is incorporated into the said composition while in its plastic or uncured state substantially as described."

It will be seen from these extracts, that my Amber Composition is entirely independent of and different from the India Rubber or Gutta Percha compounds, which have hitherto furnished so much food for Controversy, and those of my colleagues who have tried the amber composition for dental purposes are unanimous in giving it the preference over all others applied to the same purpose. Indeed, any dentist comparing the amber sets with those of India Rubber or Gutta Percha, will be enabled to satisfy himself about their respective merits by mere inspection.

Respectfully yours, etc.,

GEORGE DIEFFENBACH,

Surgeon Dentist, 389 Canal Street, New York.

New York, August, 1859.

